# The Nodal signaling pathway controls left-right asymmetric development in amphioxus

**DOI:** 10.1186/2041-9139-6-5

**Published:** 2015-02-17

**Authors:** Vladimir Soukup, Luok Wen Yong, Tsai-Ming Lu, Song-Wei Huang, Zbynek Kozmik, Jr-Kai Yu

**Affiliations:** Institute of Molecular Genetics, Academy of Sciences of the Czech Republic, Videnska 1083, Prague, 14220 Czech Republic; Institute of Cellular and Organismic Biology, Academia Sinica, 128 Academia Road, Section 2, Nankang, Taipei 11529 Taiwan; Institute of Oceanography, National Taiwan University, 1 Roosevelt Road, Section 4, Taipei, 10617 Taiwan

**Keywords:** Nodal signaling, Amphioxus, Left-right asymmetry, Mouth opening, Embryonic development

## Abstract

**Background:**

Nodal is an important determinant of the left-right (LR) body axis in bilaterians, specifying the right side in protostomes and non-chordate deuterostomes as opposed to the left side in chordates. Amphioxus represents an early-branching chordate group, rendering it especially useful for studying the character states that predate the origin of vertebrates. However, its anatomy, involving offset arrangement of axial structures, marked asymmetry of the oropharyngeal region, and, most notably, a mouth positioned on the left side, contrasts with the symmetric arrangement of the corresponding regions in other chordates.

**Results:**

We show that the Nodal signaling pathway acts to specify the LR axis in the cephalochordate amphioxus in a similar way as in vertebrates. At early neurula stages, Nodal switches from initial bilateral to the left-sided expression and subsequently specifies the left embryonic side. Perturbation of Nodal signaling with small chemical inhibitors (SB505124 and SB431542) alters expression of other members of the pathway and of left/right-sided, organ-specific genes. Upon inhibition, larvae display loss of the innate alternation of both somites and axons of peripheral nerves and loss of left-sided pharyngeal structures, such as the mouth, the preoral pit, and the duct of the club-shaped gland. Concomitantly, the left side displays ectopic expression of otherwise right-sided genes, and the larvae exhibit bilaterally symmetrical morphology, with duplicated endostyle and club-shaped gland structures.

**Conclusions:**

We demonstrate that Nodal signaling is necessary for establishing the LR embryonic axis and for developing profound asymmetry in amphioxus. Our data suggest that initial symmetry breaking in amphioxus and propagation of the pathway on the left side correspond with the situation in vertebrates. However, the organs that become targets of the pathway differ between amphioxus and vertebrates, which may explain the pronounced asymmetry of its oropharyngeal and axial structures and the left-sided position of the mouth.

**Electronic supplementary material:**

The online version of this article (doi:10.1186/2041-9139-6-5) contains supplementary material, which is available to authorized users.

## Background

Bilaterians exhibit varying degrees of asymmetry along the left-right (LR) body axis. Most animals exhibit a symmetrical outward appearance with conserved directional asymmetry of the visceral organs, while others, such as snails and crabs, possess strikingly asymmetrical external features [1–3]. Many embryos usually undergo an initial symmetry-breaking event, which is followed by the asymmetrical propagation of signaling cues and gene expression, and eventually asymmetrical organ formation [4]. Although the nature of the symmetry-breaking event varies between different animals [5–7], the signaling cascade that patterns the LR axis seems to be highly conserved [1, 2, 8].

Nodal, a transforming growth factor beta (TGF-β) superfamily factor, has been previously identified to be central to determining LR asymmetry through unilateral activation of downstream genes. In mouse, *Nodal* is initially expressed bilaterally around the node, where the encoded protein interacts with its co-ligand GDF1. The Nodal/GDF1 heterodimer exhibits higher activity than the Nodal homodimer and also acts at a longer range [9]. The action of the Nodal inhibitor Cerl2 on the right side ensures that Nodal becomes preferentially active on the left side, and this activity is transferred to the left lateral plate mesoderm [10–12]. Here, Nodal activates its own expression and also triggers expression of the TGF-β factor *Lefty2* and transcription factor *Pitx2*. Lefty2, which diffuses at a high velocity and inhibits Nodal, ensures that Nodal becomes restricted to the left side; here, Nodal further enhances its own expression and expression of *Lefty2* and *Pitx2*[13]. Pitx2 subsequently triggers expression of downstream targets and promotes tissue-specific proliferation and differentiation, leading to asymmetrical development of the affected organs ([14] and literature therein).

Nodal signaling has been identified in most deuterostomes, where it determines both internal organ asymmetry and asymmetric external development [14–18]. In protostomes, Nodal signaling directs shell coiling in snails and probably also LR asymmetry of internal organs in other lophotrochozoans; on the other hand, Nodal has not been identified in ecdysozoans, despite numerous examples of directional LR asymmetries in these organisms [19–21]. Interestingly, the recent finding that the Nodal-Pitx cascade is responsible for asymmetric budding and branching morphogenesis of polyps in *Hydra* suggests that regulation of asymmetric morphogenesis by the Nodal pathway is an ancient trait that originated prior to the split of cnidarians and bilaterians [22]. Despite its conserved use throughout eumetazoans, there is a key difference in the site of expression and function of Nodal: it defines the left side in vertebrates and non-vertebrate chordates, but the right side in non-chordate deuterostomes and lophotrochozoans [15, 20]. This change of expression is likely related to the proposed inversion of the dorso-ventral axis in the common ancestor of chordates [23–26], which caused a concomitant flipping of the right and left sides.

Amphioxus is advantageous for studying the events that occurred just after the dorso-ventral inversion. This group of marine invertebrates shares many common features (including notochord, dorsal nerve cord, pharyngeal gill slits, and metameric somital segments) with vertebrates, but lacks the vertebral column and the elaborate head structures derived from neural crest cells. Several vertebrate organs have identifiable homologs in amphioxus. The early developmental stages and adult stages of amphioxus are also highly similar to their counterparts in vertebrates. Upon sequencing the whole genome [27, 28], the phylogenetic positioning of amphioxus at the earliest diverging chordate clade is supported, while vertebrates and the highly derived tunicates are now placed together as a sister group [29, 30]. Its phylogenetic position among the chordates and similarities to vertebrates have enabled amphioxus to provide crucial insights into the ancestral state of vertebrate traits [31].

**Figure 1:**
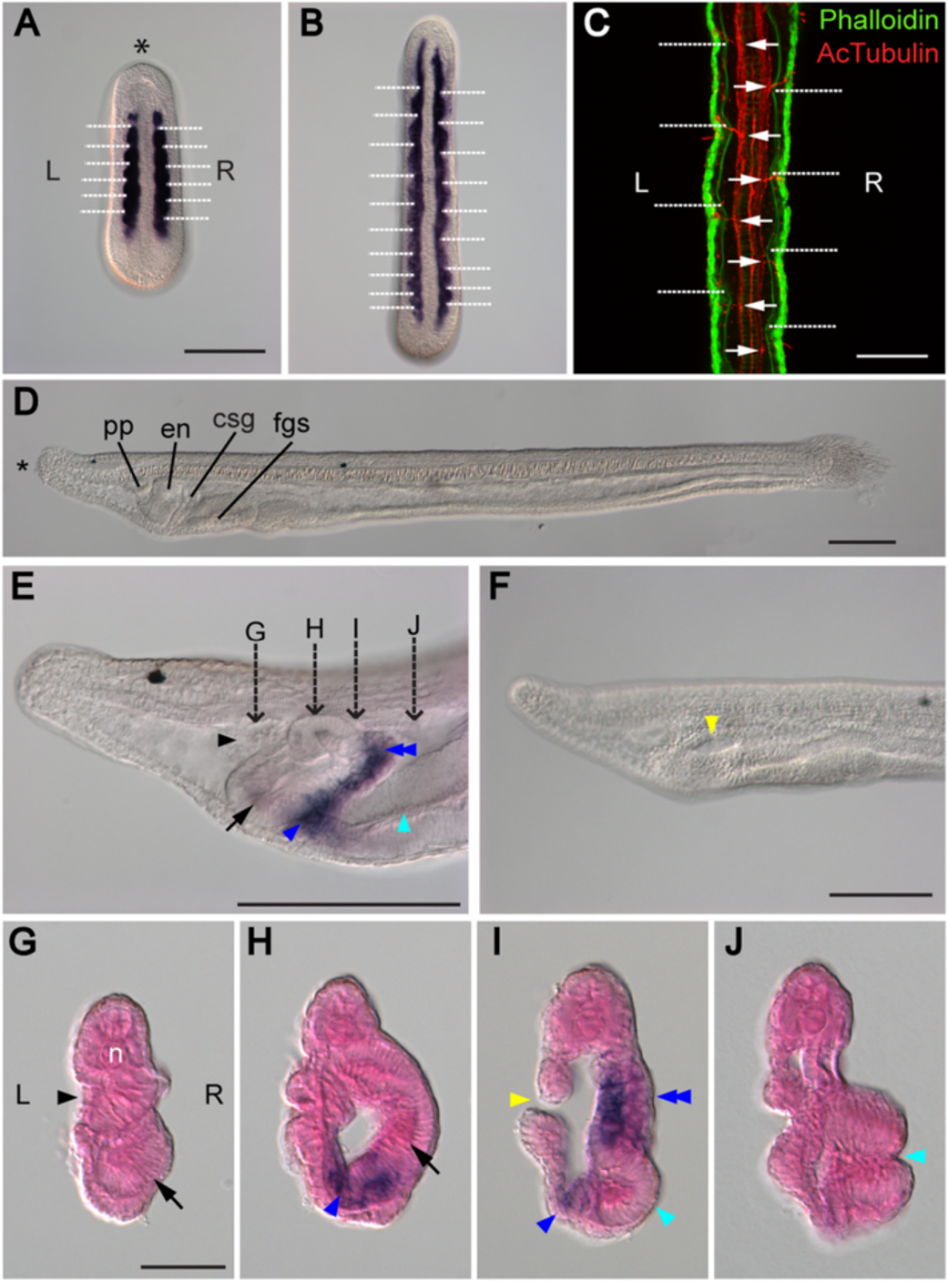
**The left-right asymmetric characters of amphioxus. (A, B)** Dorsal views of mid-neurula (N2) and late neurula (N3), marked with *m-actin* to display asymmetric arrangement of somites. White dotted lines mark the boundary of each somital segment. The asterisk (*) marks the anterior; ‘L’ represents the left side and ‘R’ the right side of the embryo. Scale bar, 100 μm. **(C)** The myomeres (phalloidin staining) and axon bundles (acetylated α-tubulin staining) of amphioxus larva (L2) are positioned asymmetrically. White dotted lines mark myomere boundaries, and white arrows mark connections of peripheral axon bundles to the neural tube. Scale bar, 50 μm. **(D)** Left lateral view of L2-stage larva. Scale bar, 100 μm. en, endostyle; csg, club-shaped gland; fgs, first gill slit; pp, preoral pit. **(E)** Left lateral view of L2-stage larva focused on the right side. The club-shaped gland is marked with *FoxE4* riboprobe. Dashed arrows mark the section planes in (G-J). Pharyngeal organs are labeled as described for G–J. Scale bar, 100 μm. **(F)** Left lateral view of L2-stage larva focused on the left-sided mouth opening (yellow arrowhead). Scale bar, 100 μm. **(G-J)** Sections of L2-stage larva from (E) showing asymmetrical positioning of pharyngeal organs. ‘n’ marks the notochord and the sections are seen from the posterior direction. The preoral pit (G, black arrowhead) and the mouth (I, yellow arrowhead) open on the left. The endostyle (H, black arrow) forms on the right. The dorsal part of the club-shaped gland (I, blue double arrowhead) is situated on the right, while its ventral part (duct of the club-shaped gland) extends to the left side (H, I, blue arrowhead). The first gill slit (I, J, cyan arrowhead) opens to the right side. Each section is 10 μm thick. Scale bar, 25 μm.

Amphioxus LR asymmetry is a peculiarity among other chordates and represents an interesting area of study (Figure 1). During embryonic development, the somites are formed asymmetrically on the left and right sides [32]; furthermore, the arrangement of somites is staggered, with the left set of somites positioned slightly forward as compared to the right set (Figure 1A,B). As a consequence, in larval and adult amphioxus, the muscle segments and peripheral nerves running along the myomere boundaries are out of register, with the left side positioned half a segment anterior to the right side [33] (Figure 1C). Even more conspicuously, the entire pharyngeal region displays a marked asymmetry (Figure 1D,E,F,G,H,I,J). The left-sided positioning of the mouth during the larval stages is considered unique to amphioxus (Figure 1F) and raises serious questions regarding the proposed homology with the median mouths of other chordates. On the left side, the structure related to the vertebrate anterior pituitary, the preoral pit, develops from the left coelomic pouch that fuses with the epidermis anterior to the mouth [34] (Figure 1E,G). On the right side, the pharyngeal wall differentiates into the endostyle (Figure 1E,H) that represents the homolog of the vertebrate thyroid gland and into the club-shaped gland, an enigmatic structure with no clear counterpart in other animals. The club-shaped gland forms a transverse tube, the dorsal secretory part of which connects to the right pharyngeal wall, while the ventral non-secretory duct opens externally on the left side [35] (Figure 1E,H,I). The pharyngeal asymmetry is further exaggerated by the first few gill slits that develop ventrally on the right side behind the club-shaped gland (Figure 1E,J). Although the marked LR asymmetric features of amphioxus have been known since the late 19th century (reviewed in [1]), it is still not completely clear whether this asymmetry is also controlled by the conserved Nodal signaling cascade. During the last decade, the expression patterns of homologs of vertebrate *Nodal*, *Lefty2*, *Gdf1*, *Cerl2*, and *Pitx2* have been described, and the LR asymmetric expression patterns of those genes hint that Nodal signaling may play a role in patterning the LR axis in amphioxus [36–41]. However, functional studies of the Nodal pathway during LR patterning have not been carried out in the amphioxus system to date.

In this study, we describe asymmetric expression of members of the Nodal signaling cascade and other putative downstream factors that are likely involved in establishing the LR asymmetry of the amphioxus body. Following the application of chemical inhibitors against Nodal signaling during amphioxus embryogenesis, we observed loss of expression of left-sided genes and ectopic expression of right-sided genes on the left side. Upon Nodal inhibition, the larvae exhibit a bilaterally symmetrical phenotype with duplication of the right-sided structures and suppression of left-sided morphology, involving loss of the mouth opening and preoral pit and loss of the offset arrangement of paraxial structures. Our findings indicate that Nodal signaling is necessary for the induction of asymmetric expression of downstream genes and for the formation of profound asymmetry of the amphioxus body.

## Methods

### Identification of left-right asymmetric gene markers in amphioxus

The sequences of *Branchiostoma floridae* genes reported to be asymmetrically expressed along the left and right sides (including *Gdf1/3*[38], *Pitx*[36, 39], *Nkx2.1*[42], *Hand*[43], *m-actin*[44], and *Hu/Elav*[33, 45]) were BLAST (Basic Logical Alignment Search Tool)-searched against the *B. floridae* expressed sequence tag (EST) database [46] (http://amphioxus.icob.sinica.edu.tw/) to identify potential cDNA clones (Additional file 1: Table S1). The clones were amplified using EST-specific primers (Additional file 2: Table S2) to synthesize probes for RNA *in situ* hybridization. Clones of *Nodal*, *Lefty*, and *FoxE4* were described previously [40, 41, 47]. *B. floridae Cerberus* cDNA, which could not be identified in the EST database, was amplified by polymerase chain reaction (PCR) using a cDNA library constructed in the pBluescript vector [48]. PCR was performed using the Expand High Fidelity^PLUS^ PCR System (Roche, Basel, Switzerland). PCR products were then ligated into the pGEM^®^-T Easy vector (Promega, Madison, WI, USA), amplified, and sequenced.

Published sequences of amphioxus genes that were previously shown to display asymmetric expression in the pharynx (*Pitx*, *Lhx3*, *Dkk1/2/4*, *FoxE4*, *FoxQ1*, and *Nkx2.1 (TTF-1)*[36, 39, 42, 47, 49–53]) were BLAST-searched for their potential *Branchiostoma lanceolatum* orthologs using the European amphioxus transcriptome database [54]. After confirmation using translated nucleotide BLAST (tBLASTx), the candidate sequences were amplified using AccuPrime *Pfx* DNA Polymerase (Life Technologies, Carlsbad, CA, USA) and sequence-specific primers (see Additional file 2: Table S2). The PCR products were cloned into the pCR-Blunt II-TOPO vector (Life Technologies, Carlsbad, CA, USA), and the identity of the clones was confirmed by sequencing. The *B. lanceolatum Krox* clone was kindly provided by Stephanie Bertrand (Laboratoire Arago, Banyuls-sur-Mer, France).

### Animal collection

Three species of amphioxus, *Branchiostoma floridae*, *Branchiostoma belcheri*, and *Branchiostoma lanceolatum*, were used in this study. *B. floridae* adults were collected in Tampa Bay, FL, USA, during the summer breeding season. *B. belcheri* adults were collected from Kinman Island near the Xiamen area in southeastern China [55]. Gametes were obtained by electric stimulation [56] or by spontaneous spawning of gravid animals. Fertilization and culturing of the embryos were carried out as previously described [56]. *B. lanceolatum* adults were collected in Banyuls-sur-Mer, France, prior to the summer breeding season and raised in the lab until spawning. The spawning of males and females was induced by temperature shift as described [57]. Embryos were staged according to Hirakow and Kajita [58, 59], and neurula-stage embryos were further divided into more defined stages according to Lu et al. [33].

### *In situ*hybridization, immunostaining, cryosection, and image acquisition

To synthesize riboprobes, cDNA fragments were amplified as templates as previously described [60]. Antisense or sense digoxigenin (DIG)-labeled riboprobes were synthesized using DIG RNA labeling mix (Roche, Basel, Switzerland) with T7 or SP6 RNA polymerase (Promega, Madison, WI, USA), depending on the insert orientation. Whole-mount *in situ* hybridization on amphioxus embryos was performed as previously described [61] with slight modifications to improve the results. Probe incubation during the hybridization process was performed at 65°C overnight. Immunostaining of F-actin and acetylated alpha-tubulin was carried out as previously described [33], with slight modifications. Embryos were de-ciliated by using a p1000 pipette to gently pipette seawater onto embryos placed in a 45-μm meshed basket. The streaming seawater pushes the embryo against the meshed surface repeatedly, which creates a force that makes the surface cilia fall off. After this procedure, embryos were fixed with 4% of PFA in MOPS buffer. The secondary antibody used to detect acetylated alpha tubulin was changed to Alexa-635-conjugated goat anti-mouse antibody. Hoechst 33342 (Invitrogen, Carlsbad, CA, USA) was used for nuclear staining. Cryosections were obtained using a Leica CM1950 cryostat (Leica Biosystems, Heidelberg, Germany) after *in situ* hybridization.

Images of embryos were taken using a Zeiss Axio Imager A1 microscope with a Zeiss AxioCam MRc CCD camera (Carl Zeiss, Jena, Germany) or using a Nikon Diaphot 300 microscope (Nikon Corporation, Chiyoda, Tokyo, Japan) with a Canon EOS 1100D camera (Canon, Inc., Chichibu, Saitama, Japan). Immunofluorescence images were taken with a Leica TCS-SP5-AOBS confocal microscope (Leica Microsystems, Wetzlar, Germany). ImageJ and Leica LAS AF were used to minimally adjust the brightness of photographs and to construct montage images of the whole larvae from multiple photographs. Imaris x64 program (version 8.0.1) was used to produce 3D reconstructions of the pharyngeal morphology of *B. floridae* larvae, with Hoechst 33342 nuclear staining acting as the template. Z-sections (section thickness: 1.968 μm; stepsize: 0.8 μm) of larvae were imaged from the dorsal side; a Leica TCS-SP5-AOBS multiphoton beam with an excitation wavelength of 810 nm was used to increase the resolution of each z-stack. Overall larval morphology was created using the automated function, with the nuclear signal on each layer acting as the template. Whole mount larval morphology was then turned transparent, while the pharyngeal region was selected out by manipulating the intensity threshold and voxel value. The preoral pit, endostyle, club-shaped gland, and gill slits were contoured manually by selecting the outline of the nuclear staining of each organ across each z-section from the dorso-ventral plane. To ensure that the manually contoured structures accurately depicted the relevant organs, an automated contour of the whole pharyngeal region was generated; the automatic and manual contours were cross-checked to ensure they overlapped. Further cross-checking with previous cryosections was conducted to ensure consistency of the model. The outline of the mouth was generated by removing the rest of the automated pharyngeal region model with several clipping planes, leaving the surface around the mouth opening. Snapshots and videos of the 3D model were also made in Imaris x64.

### Pharmacological inhibition of Nodal signaling

*B. floridae*, *B. belcheri*, and *B. lanceolatum* embryos and larvae were treated with Nodal signaling inhibitors SB431542 (Tocris, Bristol, UK) and SB505124 (Sigma, St. Louis, MO, USA) at the indicated concentrations and for various lengths of time.

For *B. floridae* and *B. belcheri* treatments, SB505124 and SB431542 were dissolved in dimethyl sulfoxide (DMSO) to prepare a 50 mM stock. The stock was then diluted with filtered seawater to make working solutions of 5 and 10 μM for SB505124 and 10 and 20 μM for SB431542. The embryos were treated at the late gastrula stage (G5/6) by direct application. The control group was treated with equal amounts of DMSO. The embryos were then collected and fixed at early pre-hatching neurula (N0), early neurula (N1), mid-neurula (N2), late neurula (N3), and open mouth (L2) stages for further analysis.

For *B. lanceolatum* treatments, SB505124 was dissolved in DMSO to prepare a 3 mM stock. This stock solution was diluted in filtered seawater to final concentrations of 0.1, 0.5, 1.0, 5, 10, and 50 μM and applied to amphioxus embryos at N0. The larvae were raised until the open mouth stage and then fixed. Control embryos were treated with filtered seawater containing an equal amount of DMSO. Larvae treated with a concentration of 0.1 μM displayed a wild-type phenotype while larvae treated with 0.5 μM or higher showed a phenotype with altered morphology. After this pilot experiment, we performed two types of treatments. For long-term treatments, embryos were raised in 1 μM SB505124 from cap-shaped gastrula (G3), mid-gastrula (G4), late gastrula (G5/G6), gastrula/neurula (N0), hatching neurula (N1), mid-neurula (N2), or early larva (L1) until the open mouth stage (L2) and then fixed. For time-restricted treatments, embryos at G4, G5, N0, or N1 were treated with 0.5 μM SB505124; the concentration was adjusted to 0.1 μM by adding the adequate amount of filtered seawater at N0, N1, N2, N3, or L1. Embryos were then raised until L2 and fixed. After fixation, the control and treated larvae were used for morphological analysis and *in situ* hybridization.

## Results

### Asymmetric expression of developmental regulatory genes across the left-right axis during amphioxus embryogenesis and larval development

**Figure 2:**
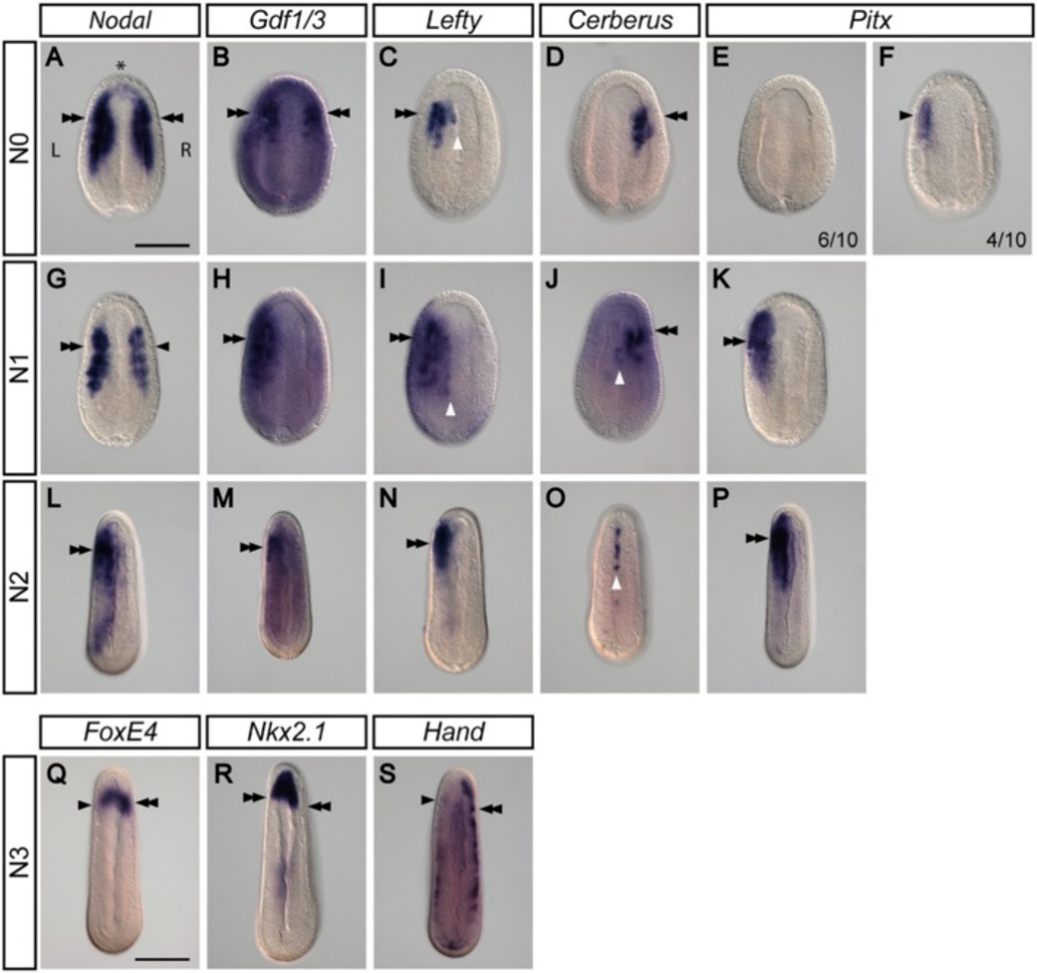
**Left-right asymmetric expression of developmental regulatory genes during amphioxus embryogenesis.** All images are of *B. floridae*, taken from the dorsal view. ‘L’ marks the left side, ‘R’ marks the right side, and the asterisk (*) marks the anterior. Double arrowheads indicate stronger expression patterns, and single arrowheads indicate relatively weaker expression patterns. White arrowheads indicate the midline. Scale bar, 100 μm. **(A-F)** At the onset of neurulation (stage N0), *Nodal* and *Gdf1/3* are expressed symmetrically on both the left and the right sides of the embryo. *Lefty* is expressed on the left side, while *Cerberus* is expressed on the right side; these are the first signs of asymmetrical gene regulation. *Pitx* is expressed on the left side of a fraction of embryos, suggesting that *Pitx* is just starting to be transcribed. **(G-K)** At the early neurula stage (N1), *Nodal* becomes expressed preferentially on the left side, while *Gdf1/3*, *Lefty*, and *Pitx* are expressed exclusively on the left side. Additionally, *Lefty* is also expressed at the midline. *Cerberus* transcripts are found on the right side and also at the midline. **(L-P)** At the mid-neurula stage (N2), *Nodal*, *Gdf1/3*, *Lefty*, and *Pitx* are expressed exclusively on the left side while *Cerberus* is expressed at the midline. **(Q-S)** At the late neurula stage (N3), some organ-specific genes already display asymmetric expression. Expression of *FoxE4*, *Nkx2.1*, and *Hand* is biased towards the right side.

To establish whether cephalochordates use a conserved genetic network for LR patterning, we first surveyed the expression patterns of amphioxus homologs of the Nodal signaling cascade and genes known to be responsible for vertebrate LR asymmetric development. Previously, it has been shown that *Nodal*, *Gdf1/3*, *Lefty*, and *Cerberus* are all expressed symmetrically in the dorsal mesendoderm during amphioxus gastrulation [37, 38, 40, 41]. To further determine the exact starting point of LR asymmetric expression of these important signaling molecules, we carefully selected finely staged *B. floridae* neurulae for examination. At the N0 stage (neurulae with zero somites), *Nodal* and *Gdf1/3* are expressed bilaterally in the dorsal paraxial mesoderm (Figure 2A,B). Interestingly, *Lefty* expression becomes localized to the left side up to the embryonic midline (Figure 2C). On the other hand, the Nodal antagonist *Cerberus* is expressed only on the right side at this stage (Figure 2D). Thus, left-sided *Lefty* expression and right-sided *Cerberus* expression appear to represent the earliest recognizable LR asymmetric gene expression during amphioxus embryogenesis. In addition, *Pitx* expression cannot be detected in six of the ten N0-stage embryos examined (Figure 2E); in the remaining four N0-stage embryos, *Pitx* is expressed weakly on the left side (Figure 2F), suggesting that left-sided *Pitx* expression has just begun to be established at the N0 stage.

At the N1 stage (neurulae with three somites), Nodal is still expressed in a bilateral fashion, but the expression in the left domain is stronger than that in the right domain (Figure 2G). Similarly, the expression domains of *Gdf1/3* and *Lefty* are more restricted to the left side by this stage (Figure 2H,I); *Lefty* transcripts are also found medially up to the embryonic midline (Figure 2I, white arrowhead). *Cerberus* is expressed on the right side of the embryo (Figure 2J), and some *Cerberus* transcripts can be detected at the midline (Figure 2J, white arrowhead). At this stage, *Pitx* is strongly expressed on the left side in both the mesendoderm and the ectoderm (Figure 2K).

At the N2 stage (neurulae with eight somites), *Nodal*, *Gdf1/3*, and *Lefty* transcripts are found mainly on the left side (Figure 2L,M,N). *Cerberus* expression is no longer observed at the right side, but instead is located in the midline (Figure 2O). *Pitx* expression remains on the left side in the anterior region of the embryo (Figure 2P). In addition to the aforementioned Nodal cascade genes, several transcription factor genes also display the LR asymmetric expression pattern during this stage and the subsequent N3 stage (neurulae with more than eight somites). *FoxE4*, a gene specifically expressed in the developing club-shaped gland and the adult endostyle [47, 62], is preferentially expressed on the right side (Figure 2Q). *Nkx2.1* is expressed in the anterior archenteron, with right-sided expression reaching slightly posterior to the expression on the left side (Figure 2R). The *Hand* gene is expressed in the lateral/ventral mesoderm during amphioxus development [43], and its expression is consistently stronger on the right side as compared to that on the left side (Figure 2S).

**Figure 3:**
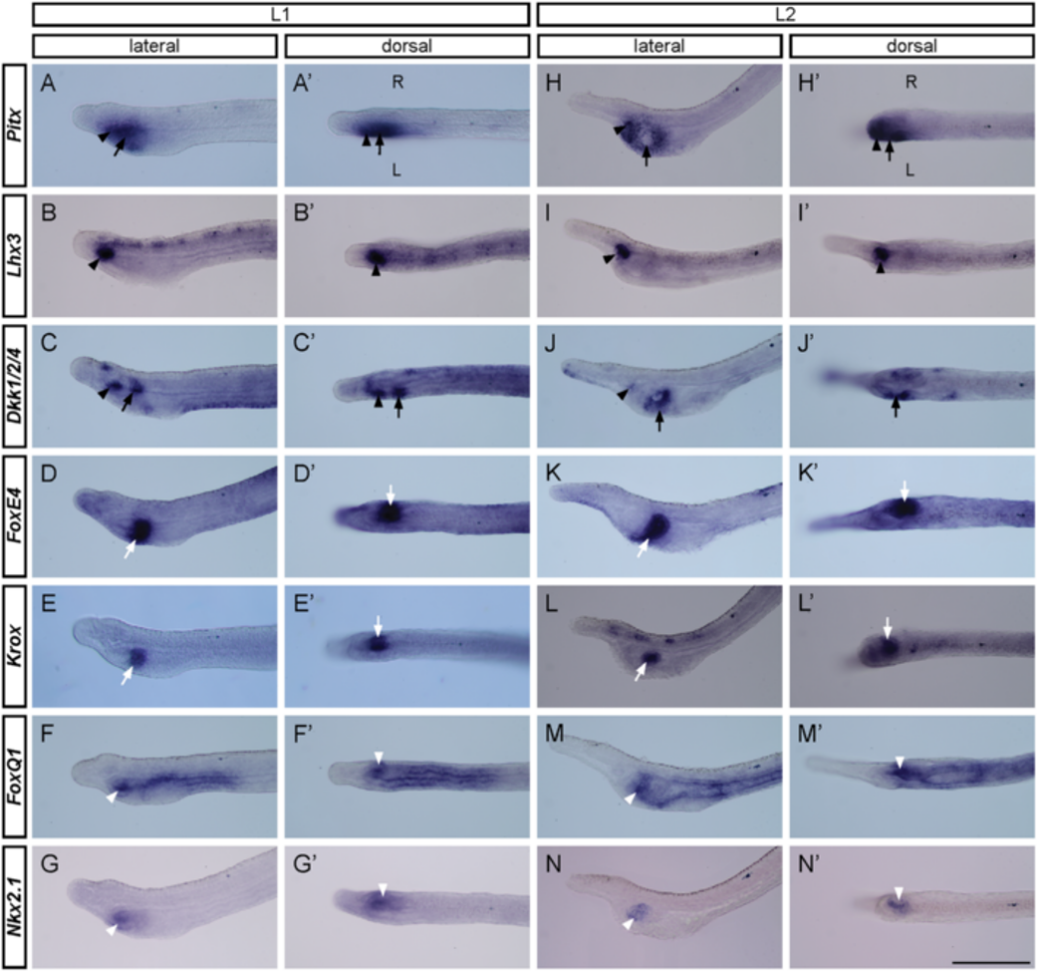
**Left-right asymmetric expression of developmental regulatory genes during larval stages.** Images are of *B. lanceolatum* at the early larval stage (L1) and the open mouth stage (L2) from either left lateral view or dorsal view. ‘L’ marks the left side and ‘R’ marks the right side; anterior is to the left. Black arrowheads indicate the preoral pit, black arrows mark the mouth region, white arrows point to the club-shaped gland, and white arrowheads mark the endostyle. Scale bar, 100 μm. **(A-G, A’-G’)** At L1, *Pitx* is expressed broadly in the left anterior pharyngeal region spanning the area of the prospective preoral pit and the mouth. *Lhx3* expression is confined to the prospective preoral pit, while *Dkk1/2/4* is expressed focally in the regions destined to form both the preoral pit and the mouth. *FoxE4* and *Krox* transcripts are restricted to the prospective club-shaped gland, where the expression pattern of *Krox* is nested within that of *FoxE4. FoxQ1* is expressed anterior to *FoxE4* in the prospective endostyle region and also at other regions within the pharynx. *Nkx2.1* transcripts are confined solely to the endostyle region. **(H-N, H’-N’)** The L2 stage exhibits well-developed pharyngeal structures, with expression patterns similar to those at L1. *Pitx* and *Dkk1/2/4* continue to be expressed in the preoral pit and the mouth, while *Lhx3* expression is confined solely to the preoral pit. *FoxE4* and *Krox* are expressed in the club-shaped gland, with *FoxE4* marking the whole structure, while *Krox* transcripts are confined to its dorsal part. In addition to its expression elsewhere in the pharynx, *FoxQ1* is co-expressed with *Nkx2.1* in the endostyle.

Major organogenesis starts to take place during the subsequent larval stages. At the L1 stage, the larva exhibits LR asymmetric expression of several genes in the pharyngeal region. Here, we show *B. lanceolatum* images as representatives in Figure 3, because during the course of this study, we obtained more pharyngeal gene markers from *B. lanceolatum*. Available gene expression patterns from *B. floridae* show identical patterns (Additional file 3: Figure S1). On the left side, *Pitx* is expressed broadly in the anterior pharynx spanning the mouth and preoral pit areas (Figure 3A,A’), whereas *Lhx3* and *Dkk1/2/4* expression is more focused (Figure 3B,B’,C,C’). Aside from their expression in the neural tube, *Lhx3* transcripts are also found in the prospective preoral pit, while *Dkk1/2/4* is expressed superficially both in the prospective preoral pit and in the region destined to develop into the mouth opening (Figure 3B,B’,C,C’). On the right side, *FoxE4* and *Krox* are both expressed just opposite the prospective mouth opening, with the *Krox* pattern nested within the *FoxE4* pattern (Figure 3D,D’,E,E’). *Nkx2.1* and *FoxQ1* are expressed slightly anterior to the *FoxE4*-*Krox* pattern on the right side; *FoxQ1* is also expressed posteriorly along the length of the pharynx (Figure 3F,F’,G,G’).

At the L2 stage, the pharyngeal region differentiates into distinct organs, namely the preoral pit and mouth on the left side and the endostyle and club-shaped gland on the right side. On the left side, *Pitx* and *Dkk1/2/4* continue to be expressed at the margins of the mouth opening and, together with *Lhx3*, are also expressed in the preoral pit (Figure 3H,I,J,H’,I’,J’). On the right side, *FoxE4* transcripts become confined to the whole club-shaped gland (Figure 3K,K’), while *Krox* is confined to the dorsal part of this gland (Figure 3L,L’). Additionally, expression of both *Nkx2.1* and *FoxQ1* is largely restricted to the endostyle on the right side, and *FoxQ1* expression is also observed in the pharyngeal bands (Figure 3M,M’,N,N’).

In summary, we observed gradual establishment of asymmetric gene expression of (i) signaling molecules in the Nodal signaling cascade and (ii) the putative downstream transcription factor *Pitx* across the LR axis during the early neurula stage (N0 to N1). We also characterized a number of marker genes that exhibited LR asymmetric expression patterns in the mid/late neurula stage or in the subsequent larval stages. In the following sections, we will use the expression patterns of these marker genes to assess the effects of blocking Nodal signaling with small molecule inhibitors during amphioxus development.

### Treatments with Nodal signaling inhibitors disturb the expression of early left-right regulatory genes, even before morphological asymmetry can be observed

**Figure 4:**
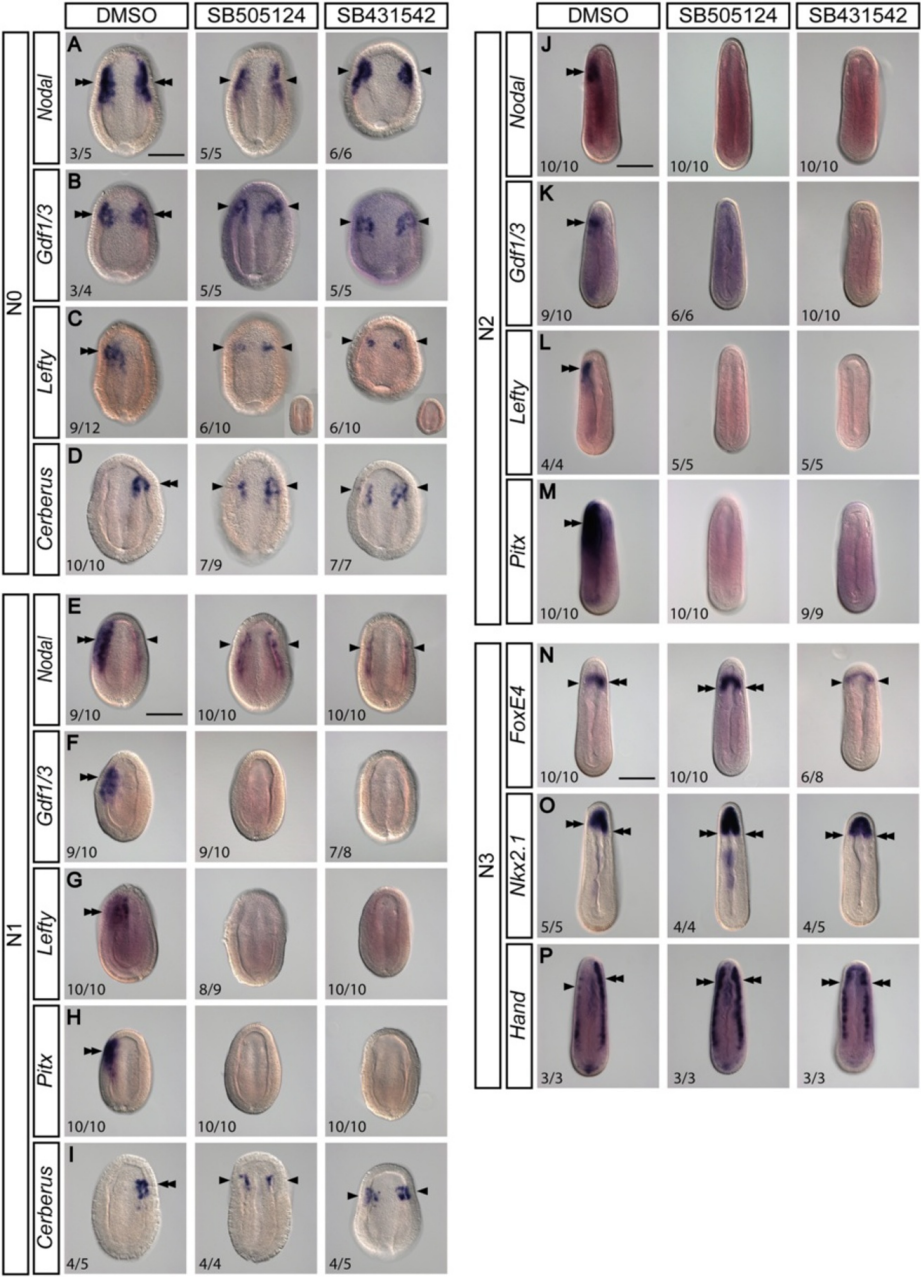
**Inhibition of the Nodal signaling pathway changes the expression pattern of left-right regulatory genes.** Images are of *B. floridae* taken from the dorsal view. Double arrowheads mark strong expression and single arrowheads mark weak expression. Scale bar, 100 μm. **(A-D)** At the onset of neurulation (N0), embryos treated with Nodal inhibitors exhibit bilateral expression of *Nodal* and *Gdf1/3*, albeit diminished as compared to controls. Similarly, the strong unilateral expression of *Lefty* and *Cerberus* in controls is converted into reduced bilateral expression by each treatment. **(E-I)** At the early neurula stage (N1), *Nodal* expression remains bilateral but is greatly diminished in the treated embryos. Expression of genes expressed on the left side (*Gdf1/3*, *Lefty*, and *Pitx*) is lost in the treated embryos, while *Cerberus* expression becomes bilaterally symmetrical. **(J-M)** At the mid-neurula stage (N2), *Nodal*, *Gdf1/3*, *Lefty*, and *Pitx* expression cannot be detected in the treated embryos. **(N-P)** At the late neurula stage (N3), the right-sided bias in expression of *FoxE4*, *Nkx2.1*, and *Hand* is abolished by the treatment, and these genes become symmetrically expressed on both left and right sides.

We next examined whether blocking Nodal signaling would affect the asymmetric expression of Nodal cascade genes and other LR marker genes. We treated *B. floridae* embryos with Nodal signaling inhibitors SB505124 and SB431542 starting from the late gastrula stage (G5/G6) and then analyzed these embryos at subsequent stages. Treated N0 embryos exhibit bilateral expression of *Nodal* and *Gdf1/3*, as compared to controls (Figure 4A,B). Expression of *Lefty* and *Cerberus*, which is restricted to either the left or right sides in control embryos, becomes bilateral for both genes upon the treatment (Figure 4C,D). The treated larvae exhibit slightly reduced expression levels of all the examined factors, and in the case of *Lefty*, the expression can even be reduced to an undetectable level (Figure 4C, inset images).

At the N1 stage, when expression of *Nodal* is normally biased to the left side, the treated embryos display weak *Nodal* expression on both sides, and the pattern appears to be symmetrical (Figure 4E). Similarly, expression of the other two left-sided genes, *Gdf1/3* and *Lefty*, is abolished by the treatment (Figure 4F,G) and expression of the downstream transcription factor *Pitx* is also lost (Figure 4H). On the other hand, treatment changes the expression pattern of *Cerberus* from right-sided to bilateral (Figure 4I). At the subsequent N2 stage, treatment abolishes expression of *Nodal* (Figure 4J). Similarly, expression of *Gdf1/3*, *Lefty*, and *Pitx* is no longer detectable in the treated N2 embryos (Figure 4K,L,M).

Analysis of other genes displaying asymmetric expression at N3 revealed that their expression patterns are also affected by treatments with Nodal inhibitors. *FoxE4*, the expression of which is biased towards the right pharyngeal wall in wild types, exhibits symmetric expression in a horseshoe-shaped pattern upon either treatment (Figure 4N). In a similar manner, the rightward-biased expression pattern of *Nkx2.1* becomes symmetrical following treatment (Figure 4O); the expression of *Hand*, which normally shows differential expression between the left and right lateral plate mesoderm, becomes equally strong on both sides (Figure 4P).

Taken together, these data demonstrate that inhibition of Nodal signaling abolishes the asymmetrical expression of the Nodal cascade genes and results in symmetrical expression of other downstream organ-specific genes during the neurula stage; therefore, Nodal signaling is necessary for establishing the LR molecular asymmetry in developing amphioxus embryos.

### Treatments with Nodal signaling inhibitors alter the left-right asymmetric arrangement of developing muscle blocks and the nervous system

**Figure 5:**
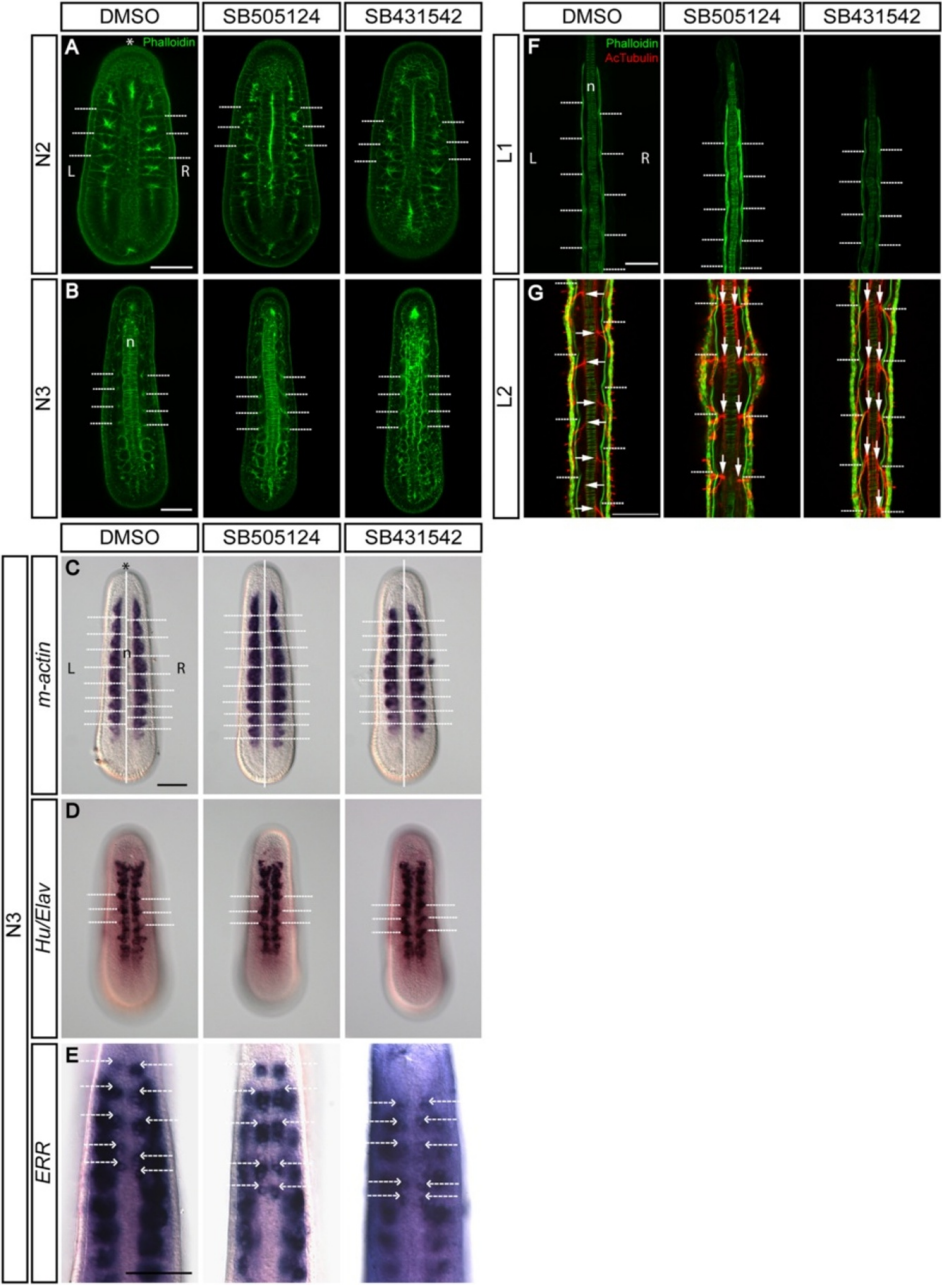
**Inhibition of Nodal signaling alters the left-right asymmetric arrangement of muscle segments and nervous system.** All images are taken from the dorsal view. ‘L’ marks the left side, ‘R’ marks the right side, and asterisks (*) mark the anterior. The notochord is marked with an ‘n’. Dashed lines mark somite borders and white arrows mark axons of the peripheral nerves. Scale bar, 50 μm. **(A, B)** *B. belcheri* embryos were stained with phalloidin to mark somite outlines. Morphological asymmetry is barely visible at N2, but asymmetrical arrangement of the somites is apparent by the N3 stage. Treatment causes the staggered arrangement of somites to become symmetrical. **(C-E)** Expression of *B. floridae m-actin* confirms the asymmetrical arrangement of the somites, and expression of *Hu/Elav* and *ERR* reveals staggered arrangement of neurons in the central nervous system. In the treated embryos, the staggered expression patterns of *m-actin*, *Hu/Elav*, and *ERR* along the anterior-posterior axis become bilaterally symmetrical. **(F-G)** Representative *B. belcheri* larvae stained with phalloidin (green) and acetylated α-tubulin (red). At larval stages, inhibition of Nodal signaling results in symmetrization of the myotomes and of axons of the peripheral nerves.

Asymmetrical somite arrangement, which occurs at the mid-neurula stage, is the earliest recognizable morphological feature of LR asymmetric development in amphioxus (reviewed in [1]). To investigate the effect of blocking Nodal signaling on the development of somite structures, we treated *B. floridae* and *B. belcheri* embryos with SB505124 and SB431542 from the late gastrula stage (G5/6) and then observed somite development in the neurula and myomere structure at larval stages using phalloidin staining of F-actin or using *in situ* hybridization of the muscle actin gene (*m-actin*). Phalloidin staining enabled detection of mild asymmetrical arrangement of somites in N2-stage neurulae, with left-sided somites positioned slightly more forward as compared to those on the right (Figure 5A, DMSO control); this asymmetrical pattern became more apparent at the N3 stage when embryos have more than eight somites (Figure 5B, DMSO control). After treatments with either Nodal inhibitors, somites become symmetrically aligned on both sides of the developing notochord at the N2 and N3 stages (Figure 5A,B). Consistently, *m-actin in situ* hybridization showed the same effect of blocking Nodal signaling on somite asymmetry (Figure 5C), that is, the *m-actin*-expressing muscle precursor cells became symmetrically aligned in the treated N3 embryos. This effect is especially apparent in the middle/posterior part of the body, where the normal asymmetric arrangement of somites is most easily discerned (Figure 5C, white dashed lines).

Previous anatomical studies have revealed the asymmetrical arrangement of amphioxus somatic motoneurons in the central nervous system (CNS) and their axonal tract structures [63–65]. Several studies on developmental gene expression patterns also uncovered such LR asymmetric patterns in putative CNS neurons during amphioxus embryogenesis [45, 66–70], indicating that the developing CNS also exhibits LR asymmetry. More importantly, this CNS neuron asymmetry corresponds to the asymmetric somite arrangement in neurula-stage embryos, suggesting that their development might be controlled by a common developmental pathway. To determine whether Nodal signaling also controls the LR asymmetrical distribution of CNS neurons, we used the expression pattern of the pan-neuronal marker *Hu/Elav* to examine the distribution of post-mitotic neurons in the embryos treated with the two Nodal signaling inhibitors. Consistent with previous descriptions, we observed that CNS neurons form recognizable clusters in two longitudinal columns along the rostral-caudal axis of the developing CNS in normal N3-stage embryos (Figure 5D, DMSO control). Much like the asymmetry of somites, the arrangement of these neuronal clusters is staggered between the left and right columns, with the left ones being positioned slightly forward of the right ones (Figure 5D, white dashed lines in DMSO control). Nodal inhibitor treatments caused the arrangement of CNS neuronal clusters to become bilaterally symmetrical (Figure 5D), consistent with the change seen in the somite development. In addition, *ERR*, which stains both somites and certain neurons in the anterior CNS [66], also exhibits asymmetrical expression along the LR axis (Figure 5E, DMSO control). Upon treatment, expression of *ERR* loses its staggered arrangement along the anterior-posterior axis and becomes bilaterally symmetrical (Figure 5E). The arrangement of somatic musculature and neurons at the N3 stage becomes more pronounced with subsequent differentiation of these tissues; by L1 and L2, the control larvae exhibit a clear alternating pattern of muscle blocks and axonal structures of spinal nerves (Figure 5F,G, DMSO controls), while the larvae treated with Nodal inhibitors display bilaterally symmetrical patterns of muscle blocks and the axon-innervated positions of the CNS (Figure 5F,G).

Overall, these data suggest that the Nodal signaling pathway is necessary for the proper development of asymmetrical muscular and neural tissues in amphioxus and that inhibition of the Nodal pathway causes symmetrization of these tissues.

### Inhibition of Nodal signaling results in the loss of the left-sided mouth opening and duplication of right-sided pharyngeal structures

**Figure 6:**
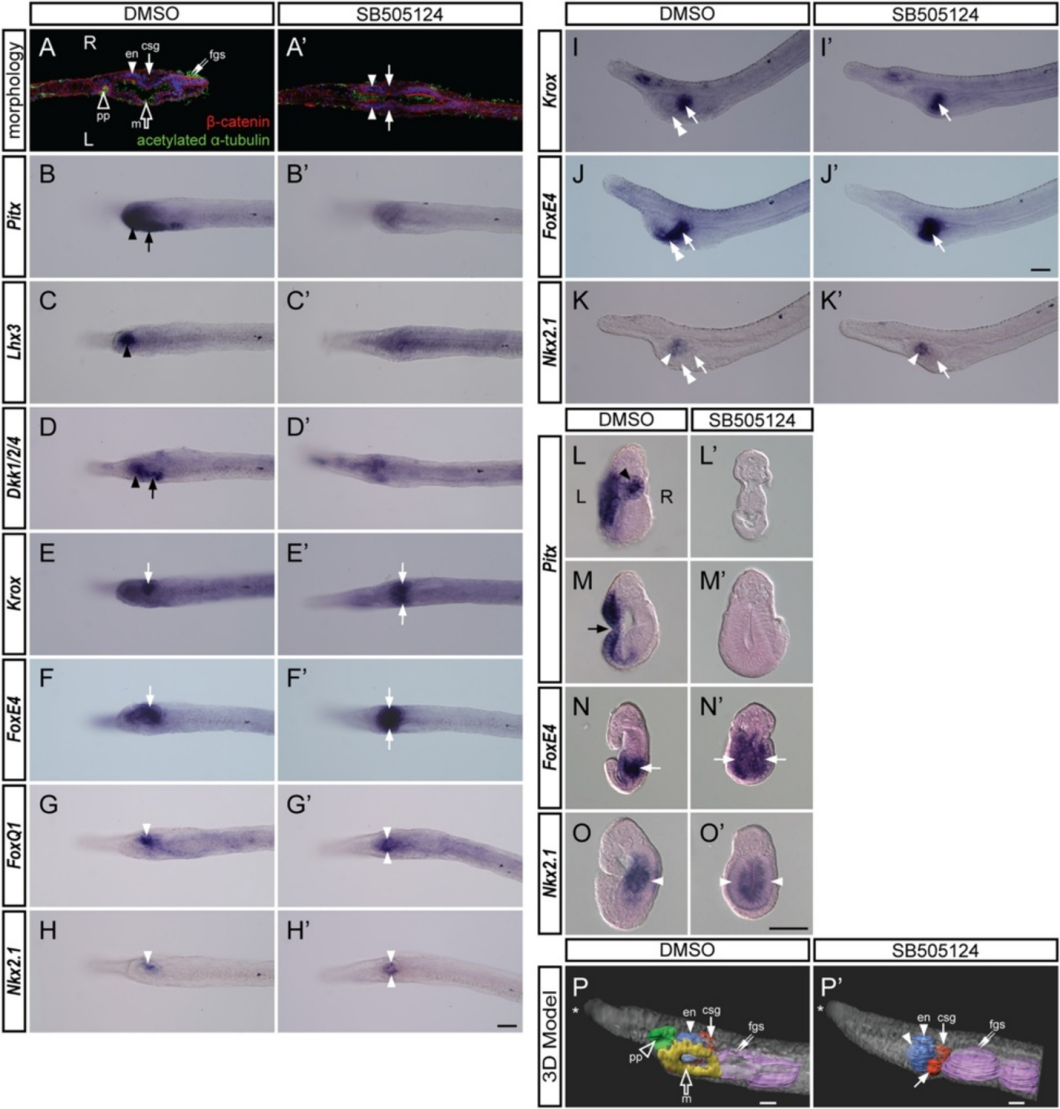
**Inhibition of Nodal signaling disrupts formation of the left-sided mouth and causes duplication of the right-sided pharyngeal structures in amphioxus larvae. (A, A’)** Dorsal views of the pharyngeal region of *B. lanceolatum* L2 larvae stained for β-catenin and acetylated α-tubulin. ‘L’ indicates the left side and ‘R’ indicates the right side; anterior is to the left. Following administration of SB505124, the larvae exhibit loss of the preoral pit (pp, black arrowhead), the mouth (m, black arrow), and the first gill slit (fgs, white double arrow) and ectopic development of the club-shaped gland (csg, white arrow) and the endostyle (en, white arrowhead). **(B-H, B’-H’)** *In situ* hybridization of the treated *B. lanceolatum* larvae revealed loss of expression of *Pitx*, *Lhx3*, and *Dkk1/2/4* and bilateral expression of *Krox*, *FoxE4*, *FoxQ1*, and *Nkx2.1* concomitant with the duplication of the right side. **(I-K, I’-K’)** View from the left side displaying loss of the duct of the club-shaped gland (white double arrowhead) upon treatment with SB505124 as deduced from the expression of *Krox*, *FoxE4*, and *Nkx2.1*. **(L-O, L’-O’)** Transverse sections of *B. floridae* larvae through the pharynx showing the loss of *Pitx* expression concomitant with the loss of both the preoral pit (black arrowhead) and the mouth (black arrow) and the ectopic left-sided expression of *FoxE4* and *Nkx2.1* concomitant with the ectopic development of the dorsal part of the club-shaped gland (white arrowhead) and the endostyle (white arrow) after treatment with SB505124. **(P, P’)** Snapshots of the 3D model of *B. floridae* larva reveal consistent morphological changes with *B. lanceolatum* larvae for both the normal and treated embryos. The snapshots are taken from the left lateral view. Asterisks (*) mark the anterior. Green, preoral pit; yellow, mouth region; blue, endostyle; red, club-shaped gland; purple, gill slits. Scale bars, 25 μm.

To investigate the effect of inhibition of Nodal signaling on the development of the amphioxus pharynx, we treated *B. lanceolatum* and *B. floridae* embryos with SB505124 from N0 to L2. We analyzed the pharyngeal morphology of the control and treated larvae using immunofluorescence and *in situ* hybridization (Figure 6 and Additional file 3: Figure S1).

Confocal microscopy analysis revealed that the pharynx of the treated larvae exhibits symmetrical morphology, with loss of left-sided structures such as the mouth and the preoral pit. Moreover, the left side of the treated larvae seems to be a mirror image of the right side, with duplication of the otherwise right-sided endostyle and club-shaped gland (Figure 6A,A’) To confirm the identity of individual pharyngeal structures, we performed *in situ* hybridization against previously identified organ-specific marker genes (see Figure 3). In agreement with the morphological analysis, *in situ* hybridization demonstrated that genes expressed in the preoral pit and in the mouth in the control larvae (*Pitx*, *Lhx3*, and *Dkk1/2/4*) become downregulated following treatment (Figure 6B,B’,C,C’,D,D’). On the other hand, *Krox* and *FoxE4* transcripts, which mark the right-sided club-shaped gland in wild type, exhibit ectopic expression on the left side of the treated larvae, thus presenting a bilaterally symmetrical signal (Figure 6E,E’,F,F’). Detailed analysis uncovered that the *FoxE4*-positive and *Krox*-negative region of the club-shaped gland (that is, the duct of the club-shaped gland) is lost in the treated larvae, suggesting that only the dorsal part of the club-shaped gland is duplicated (Figure 6I,I’,J,J’). Similarly, *FoxQ1* and *Nkx2.1*, which are expressed in the right-sided endostyle in wild type, display bilaterally symmetrical signals in the anterior portion of the pharynx upon treatment (Figure 6G,G’,H,H’,K,K’). To confirm the phenotype observed in whole mounts, we sectioned the control and treated larvae upon hybridization. The treated larvae display loss of left-sided expression of *Pitx* concomitantly with loss of the mouth and preoral pit (Figure 6L,L’,M,M’), and ectopic expression of *FoxE4* and *Nkx2.1* on the left side, suggesting that the endostyle and the club-shaped gland develop on both the left and the right sides (Figure 6N,N’,O,O’). To obtain more comprehensive images of the larval morphology, we used Imaris x64 to construct 3D organ models (Figure 6P,P’, Additional file 4: Video S1, and Additional file 5: Video S2). Snapshots of the 3D model display consistent changes of morphology for the treated larvae. The predominantly left-sided preoral pit and mouth are lost upon treatment while the predominantly right-sided endostyle and club-shaped gland form ectopically on the left side, almost forming a mirror image of their right-sided counterparts.

These data suggest that inhibition of Nodal signaling results in the loss of left-sided identity, leading to the absence of the mouth and preoral pit and duplication of the right-sided identity, with ectopic development of the endostyle and club-shaped gland on the left side.

### Morphological asymmetry is specified during early neurula stages through asymmetrical expression of downstream targets

**Figure 7:**
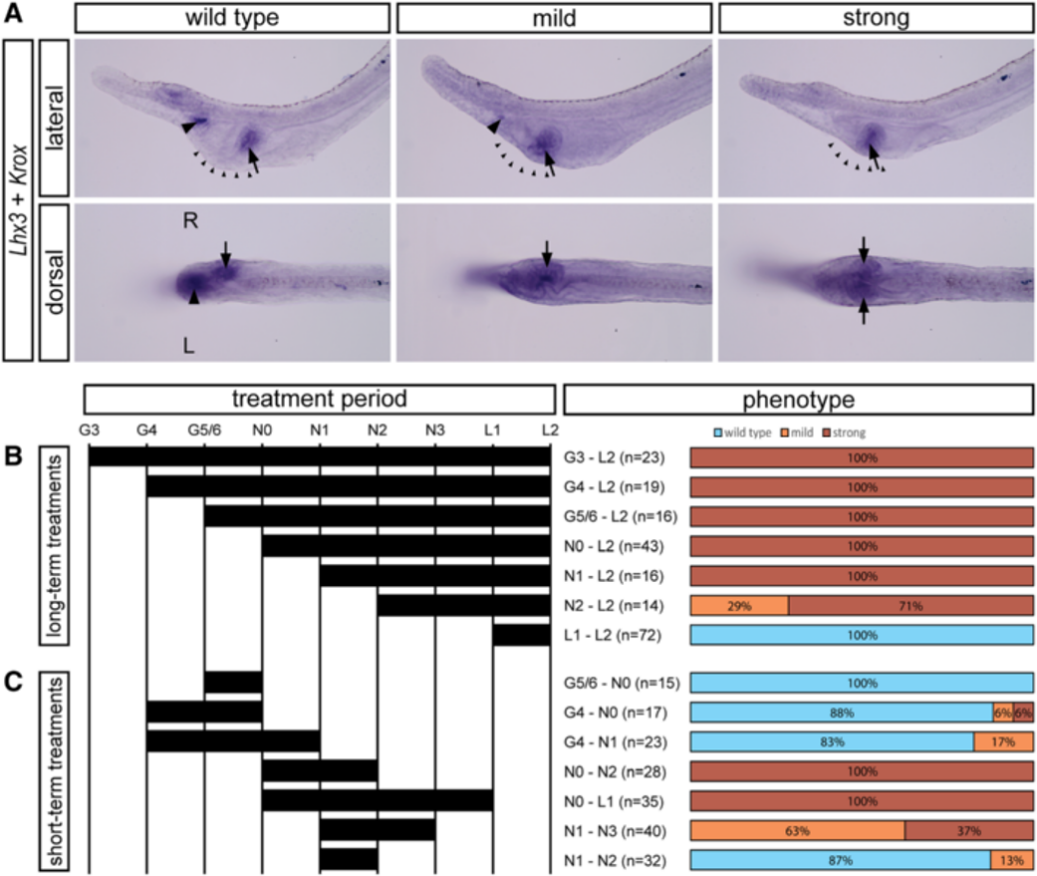
**Time-restricted inhibition of Nodal signaling reveals that LR asymmetry is specified at neurula stages. (A)** Inhibition of Nodal signaling by SB505124 results in three larval (L2) phenotypes, designated as ‘wild-type’, ‘mild’, and ‘strong’, and defined by differential expression of *Lhx3* as a marker of the preoral pit and *Krox* as a marker of the dorsal part of the club-shaped gland. The mild phenotype involves a loss of the ventral portion of the pharynx (small arrowheads) and reduced *Lhx3* expression, indicative of a reduced preoral pit (arrowhead). The strong phenotype involves a loss of the ventral portion of the pharynx (small arrowheads), complete loss of the preoral pit, and an ectopic club-shaped gland on the left side (arrow). **(B)** Long-term treatments were performed to demonstrate that Nodal inhibition during the neurula stages results in altered phenotypes. **(C)** Short-term treatments were performed to demonstrate that Nodal inhibition results in altered phenotypes, especially when administered at the N0 to N2 stages. All images are from *B. lanceolatum*.

We took advantage of the aforementioned system to determine the embryonic period during which LR asymmetry is specified. We performed a series of experiments in which SB505124 was applied to *B. lanceolatum* embryos for different time periods during development, and the resulting phenotypes at the open mouth stage (L2) were subsequently examined by *in situ* hybridization against the left-sided marker *Lhx3* and the right-sided marker *Krox*. Phenotypes were divided into three categories (Figure 7A): ‘wild type’ (asymmetrical pharyngeal morphology; expression of both *Lhx3* and *Krox* present), ‘mild’ (asymmetrical, but altered pharyngeal morphology; expression of *Lhx3* reduced or absent*,* expression of *Krox* present on the right side), and ‘strong’ (symmetrical pharyngeal morphology; expression of *Lhx3* lost, expression of *Krox* present on both left and right sides).We first performed long-term treatments, in which SB505124 was administered to amphioxus embryos at G3, G4, G5/6, N0, N1, N2, or L1 stages (Figure 7B). Application of the inhibitor at the N1 stage or earlier results in larvae with the strong phenotype, application at N2 to L2 results in larvae with either strong or mild phenotype, and application at L1 to L2 results in larvae with a wild-type phenotype. This suggests that larval morphology is most susceptible to inhibition during early neurula stages. To confirm this hypothesis, we also performed short-term treatments (Figure 7C). Application of the inhibitor during late gastrula stages does not result in a significant increase of altered morphology, and the majority of the larvae display a wild-type phenotype. On the other hand, application at early neurula stages, especially spanning N0 to N2, leads to symmetrization of the pharyngeal morphology.

These experiments are congruent with (i) the initial observation of asymmetrical expression of *Nkx2.1*, *FoxE4*, and *Hand* at N3 and (ii) the symmetrization of their expression patterns upon treatment with Nodal inhibitors (Figure 4N,O,P). Together, these data suggest that morphological asymmetry in amphioxus is specified during the neurula stages through asymmetrical expression of downstream organ-specific target genes.

## Discussion

**Figure 8:**
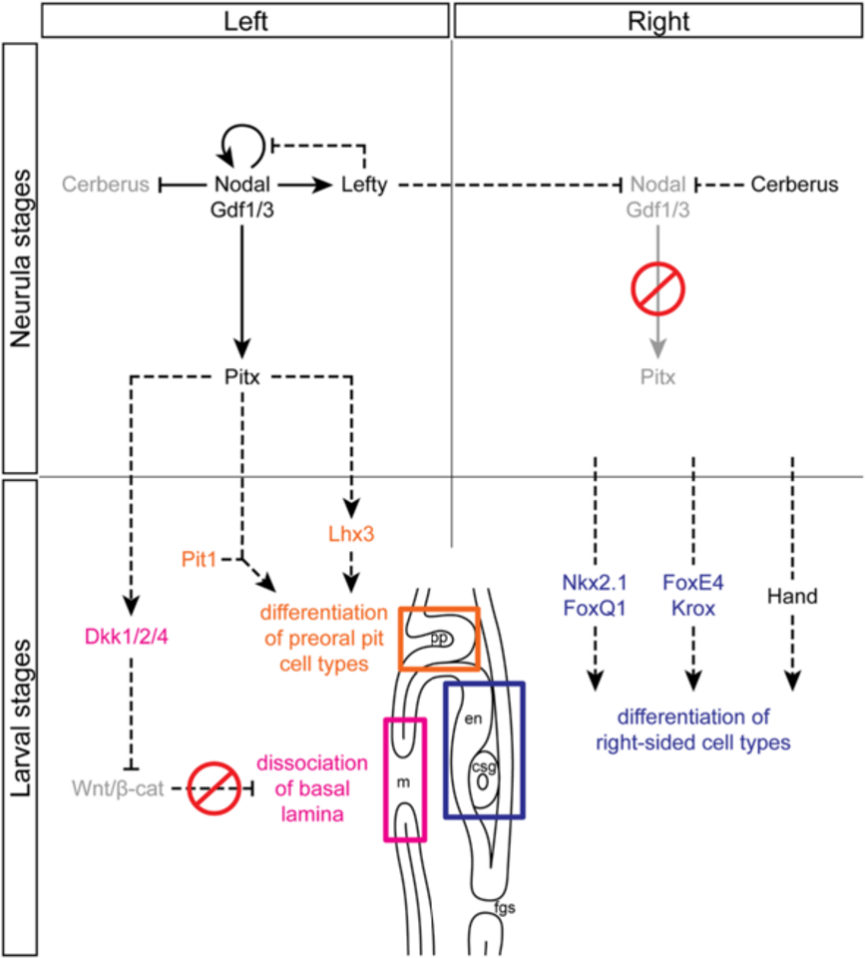
**Model of the Nodal signaling pathway during establishment of the LR asymmetry in amphioxus.** Dashed lines mark proposed interactions based on the data from other chordates. Gray text denotes inhibition of expression of the respective factors. During neurulation, *Nodal* and its co-ligand *Gdf1/3* are expressed symmetrically. However, right-sided inhibition of *Nodal* by Cerberus results in propagation of the pathway on the left side, where Nodal activates expression of *Nodal*, *Gdf1/3*, *Lefty*, and *Pitx* and inhibits expression of *Cerberus*. We propose that during the course of later development, Pitx interacts with organ-specific factors and promotes left-side specific morphogenesis. Anteriorly, Pitx is proposed to interact with Pit1 and to activate expression of *Lhx3* to promote differentiation of cell types within the preoral pit. Slightly more posterior, Pitx is proposed to regulate expression of *Dkk1/2/4*, which inhibits Wnt/β-catenin signaling, and thus promotes fusion of ectoderm and endoderm epithelia during morphogenesis of the mouth opening. The mechanism underlying activation of expression of factors responsible for right-sided morphogenesis is currently unknown. csg, club-shaped gland; en, endostyle; fgs, first gill slit; m, mouth; pp, preoral pit.

In this study, we demonstrate that expression of the Nodal signaling pathway members in amphioxus resembles that of their vertebrate orthologs and that Nodal is necessary for the left-sided expression of other downstream genes and for left-sided morphogenesis. Inhibition of Nodal results in (i) downregulation of the left-sided genes and bilateral expression of the otherwise right-sided genes and (ii) symmetrization of the larval body. Based on our detailed expression survey and time course experiments, we propose a scheme for LR axis establishment in amphioxus (Figure 8) and discuss it in an evolutionary context below.

### 1. The conservation of the role of Nodal signaling in left-right patterning in chordates

In vertebrates, the LR organizer directs the initial break of symmetry by induction of asymmetric gene expression in its vicinity. The asymmetry at the LR organizer is then transmitted to induce asymmetry in the lateral plate mesoderm, where induction of asymmetric gene expression is followed by self-enhancement and amplification of the signals that result in polarization of the body, and establishment of left and right identities. Finally, these signals target organ-specific factors that are responsible for tissue-specific morphogenesis and differentiation of left- and right-sided organs. Our study shows that the Nodal signaling pathway patterns the LR axis and specifies the left side during early amphioxus embryogenesis principally in a similar way to that observed in vertebrates (Figure 8). In amphioxus, however, Nodal acts at the place of its initial asymmetric induction, and thus, there is no transfer of asymmetrically induced signals from the organizer to the lateral plate mesoderm.

We showed that *Nodal* and its co-ligand *Gdf1/3* are expressed in a bilateral fashion at the onset of neurulation (N0) and become confined to the left side during subsequent stages, whereas Nodal inhibitors *Cerberus* and *Lefty* display early asymmetric expression. Previous studies showed that *Cerberus* expression changes from bilateral to unilateral at the gastrula/neurula transition [37]. This change resembles that of its orthologs, *Cerl2* in the mouse and *Coco* in *Xenopus*, during specification of bilateral asymmetry at the LR organizer [71–73]. The organizer houses monociliated cells, the cilia of which beat in a simultaneous manner to generate a leftward fluid flow. The flow promotes decay of *Cerl2* mRNA on the left side of the node [74], and a similar mechanism may also affect *Coco* in *Xenopus*[73]. On the left side, lack of inhibition by Cerl2 results in local activation of Nodal that further suppresses expression of *Cerl2*, whereas on the right side, expression of *Cerl2* inhibits action of Nodal [75]. Our data suggest that suppression of *Cerberus* by Nodal may also take place in amphioxus, given that inhibition of Nodal at the neurula stages results in ectopic activation of *Cerberus* expression on the left side (Figure 4D,I). Thus, although amphioxus *Nodal* and *Gdf1/3* are expressed symmetrically at N0, their right-sided suppression by Cerberus results in propagation of the Nodal signal on the left side.

Simultaneous right-sided inhibition and left-sided activation of Nodal causes a bias towards left-sided expression of the members of this pathway at the N0 to N1 transition. In vertebrates, asymmetric activity at the LR organizer is translated into asymmetry in the lateral plate mesoderm [10–12, 76, 77]. Here, members of the Nodal pathway constitute a ‘self-enhancement and lateral inhibition (SELI) system’ that reinforces determination of the left side [13]. Nodal activates its own expression and thus establishes the identity of the left side, whereas its inhibitor Lefty2 restricts Nodal activity to the left lateral plate mesoderm and prevents ectopic activation of this pathway on the right side [78]. Although no transfer of the LR signal occurs in amphioxus, the left-sided activation of the Nodal pathway at N1 is comparable to the vertebrate LR SELI system. In amphioxus, such ‘self-enhancement’ would be driven by Nodal, the inhibition of which results in the loss of expression of *Nodal*, *Gdf1/3*, *Lefty*, and *Pitx*, and leads to the loss of left-sided larval morphology. On the other hand, the ‘lateral inhibition’ would be provided by Lefty; the broad left-sided expression of this factor, which spans up to the embryonic midline, is reminiscent of the Nodal-regulating, left-sided expression of *Lefty2* and the midline expression of *Lefty1* in the mouse [79–81]. After propagation of the SELI system on the left side in amphioxus, the midline expression of *Cerberus* at N2 can further block the potential transfer and ectopic activation of Nodal signaling on the right side (Figure 2O). Our time course experiments show that normal amphioxus larval morphology is susceptible to Nodal inhibition, especially at N0 to N2, and we propose that the LR SELI system is active during this period.

The first signs of morphological asymmetry in amphioxus are observed after the LR SELI stages at N2 to N3, in which the embryos exhibit asymmetrical arrangement of somites and a unilaterally biased expression of organ-specific genes. In vertebrates, the LR morphological asymmetry is conveyed by *Pitx2*[82–87]. It is well understood that organs individually reply to the asymmetric Pitx2 signal, via differential proliferation, differentiation, cell shape changes, or apoptosis, but the molecular mechanisms that link *Pitx2* to organ-specific morphogenesis are largely unknown and are only just being deciphered (see [14] and citations therein). In amphioxus, the Nodal target Pitx probably also activates transcription of downstream factors in an organ-specific manner, thus triggering the offset placement of left-sided somites and peripheral nerves as opposed to their right-sided counterparts and promoting development of the preoral pit, mouth, and duct of the club-shaped gland (discussed later).

### 2. The significance of amphioxus left-right asymmetry and its implications for the development and evolution of the chordate characteristics

The discovery that the Nodal pathway acts to promote asymmetric development in amphioxus is in a good accord with the situation in other chordates and even non-chordate deuterostomes and other bilaterians. However, it is challenging to relate the extreme asymmetrical morphology in amphioxus to the almost perfect external symmetry in tunicates and vertebrates. Recent molecular studies support the hypothesis that a dorso-ventral inversion may have occurred in the common ancestor of chordates (reviewed in [25, 26]), although the exact mechanism leading to this inversion is still debated [23, 88–90]. Amphioxus resides within the chordate clade that split off from the lineage leading to tunicates and vertebrates just after this event. Therefore, it is difficult to determine whether the profound amphioxus asymmetry is somehow related to this event, that is, whether it reflects a recapitulation of the past evolutionary history, and thus represents a primitive chordate condition, or whether it exemplifies a peculiarity of the cephalochordate lineage with no relation to tunicates or vertebrates. The mouth plays a pivotal role in this discussion, as its presence on the left side in amphioxus larvae casts doubts on its supposed homology with the median mouth of other chordates [91].

In tunicates and vertebrates, the mouth develops at the anterior neural boundary [92, 93]. This boundary, referred to as the preplacodal ectoderm, is the location at which sensory placodes, cement organs, and the mouth originate and is demarcated by expression of members of the Pax, Six, Eya, and Dach families of transcription factors [94]. Pitx factors define the anterior-most domain within the preplacodal ectoderm and are responsible for development of the mouth, cement organs, adenohypophysis, and lens [95]. *Pitx* factors are downstream of bone morphogenetic protein (BMP) signaling (an epidermal cue) and *Otx2* (a neural cue) [96, 97]. This initial anterior expression of *Pitx* factors is distinct from later left-sided *Pitx2* expression that acts downstream of Nodal signaling and is responsible for the LR asymmetry. Additionally, both the anterior and left-sided expression of *Pitx* factors is regulated at the transcriptional level by distinct BMP (Smad1/5)- and Nodal (FoxH1)-responsive elements [87, 98, 99].

**Figure 9:**
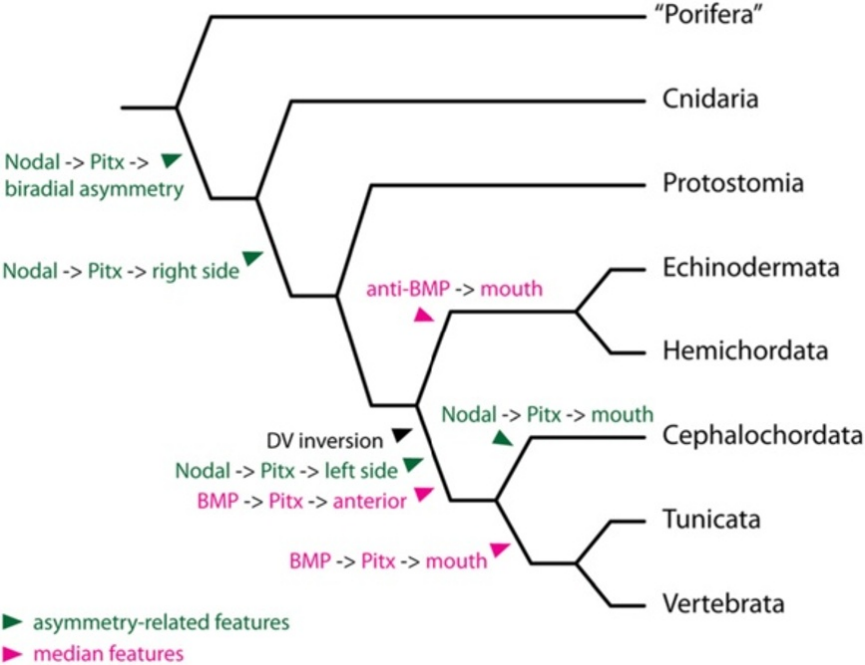
**Hypothetical scenario of the role of Pitx in LR asymmetry and mouth development across metazoans.** Green text marks LR asymmetric expression; magenta text marks median expression. In protostomes, echinoderms and hemichordates, the Nodal-Pitx pathway regulates determination of the right side. In cephalochordates, tunicates and vertebrates, on the other hand, this pathway determines the left side due to inversion of the body axes, an event that supposedly occurred at the base of chordate phylogeny. Concomitantly, new *Pitx* expression downstream of BMP signaling occurred at the anterior neural boundary (this interaction remains to be confirmed in amphioxus). Mouth development seems to be regulated differently between different deuterostome lineages. BMP signaling suppresses oral development in echinoderms and hemichordates, while it promotes oral development through Pitx in tunicates and vertebrates. In the cephalochordate amphioxus, the Nodal-Pitx pathway regulates LR asymmetry and is also responsible for development of the mouth, whose relation to the mouth of other chordates is contested.

In non-chordate deuterostomes, the mouth seems to be specified and regulated differently from that of vertebrates (Figure 9). In echinoderms, opposing Nodal/BMP signaling organizes the oral-aboral (dorso-ventral) axis, with Nodal signaling promoting, but BMP signaling inhibiting, the oral fate [100–102]. Interestingly, *Pitx2* does not seem to be part of the gene regulatory network promoting oral fate in echinoderms; if expression is observed in the oral ectoderm of an echinoderm species, it occurs only at later stages and in relation to LR asymmetry [103]. In hemichordates, the mouth develops on the non-Bmp side of the embryonic body (akin to mouth development in echinoderms), while *Pitx* expression can be found on the opposite side in the region of the prospective proboscis pore [104]. This structure has traditionally been proposed to be homologous to the anterior pituitary of vertebrates and the preoral pit of amphioxus [105]. The regulation of *Pitx* downstream of BMP signaling in hemichordates [104] is reminiscent of the situation in tunicates and vertebrates and has been proposed as a key factor of the scenario for evolutionary repositioning of the mouth. According to this scenario, the position of the mouth in the chordate ancestor changed from echinoderm- and hemichordate-like anti-BMP and non-Pitx territory to tunicate- and vertebrate-like pro-BMP and *Pitx*-expressing territory [92].

Amphioxus occupies a key position in this scenario and was previously proposed to exhibit a situation similar to that of vertebrates [92]. Yet, a *bona fide* vertebrate-like preplacodal ectoderm is not present at the anterior neural boundary [94, 106], and members of the Six, Eya, Pax, and Dach families are expressed elsewhere throughout the body [107]. Yet, *Pitx* seems to be transiently expressed at the anterior neural boundary at the late gastrula stage (G6), and this expression coincides with the partially overlapping bilateral expression of *Nodal*[39, 40]. However, this anterior *Pitx* expression is diminished at the early pre-hatching neurula stage (N0), and a new left-sided domain of *Pitx* expression is induced at the N0 to N1 transition (Figure 2E,F). Additionally, while treatments with Nodal inhibitors at neurula stages result in loss of the mouth, treatments spanning the late gastrula stages have only minor effects on mouth development (Figure 7). This suggests that either *Pitx* expression is not triggered by the Nodal signaling at these early stages or that it is not necessary for the development of the mouth. Currently, it is unknown whether the anterior *Pitx* expression is downstream of neural Otx and epidermal BMP signaling and whether this circuitry plays any role in the development of the mouth, as in other chordates. Future research should therefore be aimed at uncovering the role of anterior *Pitx* expression in amphioxus, to determine whether this feature represents a shared characteristic between tunicates and vertebrates [108] or has a deeper phylogenetic significance.

The peculiar left-sided mouth in amphioxus has long been a matter of debate as to whether or not it is homologous to the median mouth of vertebrates. Van Wijhe [109–111] argued that the amphioxus mouth is actually a modified first gill slit, which corresponds to the left spiracle of vertebrates. The presence of this left-sided opening would be advantageous for filter feeding during counter-clockwise rotation of the amphioxus larva; the original median mouth may have become greatly diminished, with its remnants present in the preoral pit. This rather controversial assumption has been contested by those who propose affiliation of the left-sided mouth of amphioxus with that of vertebrates. MacBride [112] suggested that the pharyngeal structures in amphioxus, including the mouth, are initiated in a primarily symmetrical manner and that larval pharyngeal asymmetry can be explained by differential growth within the pharynx and the resultant displacement of organs. Willey [113], on the other hand, proposed that the development of the asymmetrical pharynx in amphioxus with its left-sided mouth could be explained as an evolutionary consequence of the rostral prolongation of notochord. This prolongation would cause the pharynx to undergo a counter-clockwise torsion (when seen from behind) that would bring the anlagen of dorsal organs, like the mouth and preoral pit (see [114]), to the left side and those of ventral organs, like the endostyle and the club-shaped gland, to the right side [35, 88, 113]. Such morphogenesis could potentially be explained by differential growth or by cellular rearrangements of surrounding tissues, as proposed by MacBride [112], under the control of Nodal signaling. To date, cellular behavior during morphogenesis of the amphioxus pharynx has not been studied in depth. Holland and Holland [115] reported that cell proliferation is not correlated with potential pharyngeal torsion; rather, it is the displaced organs themselves, and not the surrounding tissues, that exhibit increased proliferation. However, if we propose that the pharyngeal organs are primarily established symmetrically and that the Nodal pathway controls the torsion, the anlagen of individual organs should remain at their supposed median position upon inhibition of this pathway (that is, the mouth and the preoral pit would be positioned dorsally and the endostyle and the club-shaped gland ventrally). In contrast, our results show that inhibition of the Nodal pathway results in the duplication of right-sided structures and does not result in the supposed development of medially positioned mouth or endostyle (Figure 6). This implies that the primordia of the pharyngeal structures are established primarily asymmetrically and that their asymmetrical arrangement is probably not a secondary consequence of the torsion.

Another option is that the mouth in amphioxus is a derived cephalochordate characteristic with no counterpart in other chordates. Yasui and Kaji [91] argued that structures associated with the mouth during amphioxus metamorphosis as well as those associated with the mouth of the larval lamprey represent traits obtained independently due to similar feeding habits and thus demonstrate analogically derived characteristics. This hypothesis, together with the findings that mouth development is regulated by medially expressed *Pitx* (downstream of BMP signaling) in tunicates and vertebrates and the sinistrally expressed *Pitx* (downstream of Nodal signaling) in amphioxus, raises serious questions regarding the *a priori* proposition of the homology of oral openings across chordates (Figure 9).

Despite the contested relationships among chordate mouths, the left-sided *Pitx* expression in amphioxus may serve similar functions as its vertebrate orthologs expressed medially at the anterior neural boundary. For example, mouse *Pitx1* and *Pitx2* regulate both early morphogenesis of Rathke’s pouch and later proliferation of pituitary cell precursors [116, 117]. Pitx1 activates expression of *Lhx3* and acts in synergy with POU1F1/Pit1 to promote differentiation of pituitary cell types [118]. Both *Lhx3* and *Pit1* homologs are expressed in the amphioxus preoral pit [52, 119], and thus, it is possible that they interact with upstream *Pitx* (Figure 8). Similarly, Pitx can regulate expression of *Dkk1/2/4* in the amphioxus mouth and preoral pit. Previously, it was shown that *Dkk1* is co-expressed with *Pitx* factors in the oral region of *Xenopus*[120, 121]. Moreover, microarray analysis demonstrated that activators of the Wnt/β-catenin pathway are downregulated, while inhibitors of this pathway, including *Dkk1*, become upregulated, in the frog oral region; additionally, *Dkk1* overexpression results in an enlarged mouth [122]. Local inhibition of the Wnt/β-catenin pathway in the mouth region is then necessary for proper dissolution of the basal lamina between the ectoderm and the endoderm and for the subsequent break of the oral membrane. A direct link between *Pitx* factors and *Dkk1* during vertebrate mouth development has not been identified at the time of writing, but Pitx2 has been shown to activate expression of *Dkk2* during development of the anterior segment of the eye in mouse [123]. The proposed regulation of *Dkk1/2/4* expression by *Pitx* and local inhibition of Wnt/β-catenin signaling by Dkk1/2/4 may thus together form an ancient mechanism to promote fusion between ectoderm and endoderm epithelia, as a prerequisite for normal morphogenesis of the amphioxus mouth and preoral pit.

We have shown here that Nodal signaling is responsible for establishment of the bilateral asymmetry of amphioxus paraxial structures, reminiscent of the role of Nodal in LR asymmetry on the pharyngeal region. The break of the initial symmetrical arrangement of paraxial structures starts at the mid-neurula stage (N2), when the fifth left somite displays a slightly anterior position and advanced differentiation to its right counterpart [124]. Shortly after (at early N3), this somite asymmetry is followed by asymmetrical development of axons of peripheral nerves [125]. During the course of development, the asymmetrical arrangement of somites and peripheral nerves is amplified, and new somites arise in a primarily asymmetric manner. Both *Nodal* and *Pitx* are expressed in the left anterior archenteron during outpocketing of the first somites (this study and [39, 40]). We have shown that inhibition of Nodal signaling results in (i) the symmetrical arrangement of somites by the late neurula stage (N3) and (ii) consequent development of bilaterally symmetrical muscle segments and peripheral nerves, resulting in the left side becoming aligned with the right side. The offset arrangement of somites regulated by Nodal signaling is specific to amphioxus development; however, the asymmetrical arrangement itself may also represent a basis for vertebrate somitogenesis. Previous experiments showed that depletion of retinoic acid in the zebrafish, chick, or mouse causes temporal acceleration of somite development on the left side [126–128], a situation reminiscent of normal development in amphioxus. Blum et al. [1] therefore proposed that retinoic acid signaling may act to shield vertebrate somites from Nodal signaling, which, in turn, may explain the vertebrate-specific transfer of the Nodal signal from the LR organizer to the lateral plate mesoderm without affecting the somites. Retinoic acid signaling thus seems to be important for buffering the lateralizing effects on vertebrate somitogenesis, while the LR alternation of somites might be a primitive feature of chordate somitogenesis.

### 3. Symmetry breaking in amphioxus: hypotheses and prospects

A further area of interest regarding LR asymmetry establishment is how the symmetry is initially broken in amphioxus. In the majority of vertebrates, symmetry breaking is associated with cilia in the LR organizer, which drive the fluid flow of certain factors to the left and thereby induce bilaterally symmetrical gene expression [5, 129]. On the other hand, there are reports that uneven distribution of ion channels during early cleavage may cause differences in pH and membrane potential between the left and right sides of embryos of certain vertebrate species, resulting in symmetry breaking [7]. Blum et al. [1] proposed that the cilia-driven flow may actually be an ancient feature dating back to the common ancestor of deuterostomes. Indeed, the dorsal wall of the archenteron of amphioxus embryo possesses monociliated cells [58, 59], and this region is reminiscent of the LR organizer in *Xenopus*[1]. Furthermore, it is worth noting that ciliogenesis occurs during the gastrula stage in amphioxus, and cilia at the outside epidermis likely start to function at very late gastrula stage (G6 to G7) to enable embryo rotation within the fertilization membrane from this stage onward [58]. However, it is unclear whether the monocilia in the dorsal wall of archenteron can also move and generate fluid flow [2]; future live-imaging studies are needed to answer this question. Nevertheless, ciliogenesis and embryonic rotation appear to commence just prior to the onset of asymmetric gene expression of *Cerberus*, *Lefty*, and *Nodal* (Figure 2), suggesting that cilia movement may play some roles in symmetry breaking in amphioxus. This hypothesis could be tested by examining the effects of blocking ciliogenesis or cilia movement on LR asymmetric development in amphioxus embryos.

It is also unclear whether uneven distribution of ion channels influences symmetry breaking in amphioxus. Although there is clear evidence for uneven distribution of maternal transcripts of germline-related genes between the first two blastomeres of the amphioxus embryo, these maternal transcripts can be deposited into either the left or the right blastomere after the first cleavage [55, 60]. It is unknown whether other maternal or early zygotic transcripts exhibit asymmetric distribution across the LR axis in cleavage-stage amphioxus embryos. Further surveys on early mRNA/protein distribution and pharmacological block of specific ion channels in cleavage-stage embryos are required to determine whether ion flux is involved in the symmetry breaking in amphioxus. We anticipate that the results obtained from amphioxus will provide important information for understanding the evolution of LR patterning mechanisms in chordates.

## Conclusions

We have shown that Nodal signaling is necessary for the establishment of LR asymmetry and for the determination of the left side of the amphioxus body. Our expression analysis and time course experiments on carefully staged neurulae allowed us to follow critical steps during the establishment of LR asymmetry; moreover, our experimental design provides a basis for future research into the existence of leftward flow-generating cilia in amphioxus and their putative role in initial symmetry breaking [1]. Given the phylogenetic position of amphioxus, such studies promise to cast light on the shared and derived character states of LR asymmetry establishment among chordates.

## Electronic supplementary material

Additional file 1: Table S1: List of clones from the EST library used to synthesize probes of *B. floridae* genes. (DOCX 42 KB)

Additional file 2: Table S2: List of PCR primers used for amplifying cDNA fragments of *B. floridae* and *B. lanceolatum* genes. (DOCX 97 KB)

Additional file 3: Figure S1: Branchiostoma floridae larvae display similar morphological changes upon treatment with SB505124. Asterisks (*) mark the anterior, ‘L’ marks the left side, and ‘R’ marks the right side. Scale bar, 25 μm. (A, A’, B, B’) *Pitx* expression marks the left-sided mouth (black arrow) and preoral pit (black arrowhead); both structures are lost upon treatment with SB505124. (C, C’, D, D’) *FoxE4* expression marks the whole club-shaped gland (white arrow), which resides mainly on the right side of the larva. Upon treatment, the club-shaped gland appears symmetrically on both the left and right sides. (E, E’, F, F’) *Nkx2.1* is expressed in the right-sided endostyle (white arrowhead). In the embryos treated with SB505124, the endostyle forms on both the left and right sides. (TIFF 9 MB)

Additional file 4: Video S1: A 3D model reconstructed from z-stack images of pharyngeal structures in a control *B. floridae* larva. The anterior is pointed towards the left side. The initial view of the larva is a lateral view, which slightly tilts towards the left side. The larva is then rotated clockwise when viewed from the posterior end. The preoral pit (green) opens on the left side, but the organ itself resides up to the midline. The position of the left-sided mouth has been indicated by highlighting the cells around it (yellow). The endostyle (blue) resides on the right side, anterior to the club-shaped gland (red); the dorsal part of the club-shaped gland is situated mainly on the right side, while the ventral part crosses over to the left. The first two gill slits (purple) reside behind the endostyle. The first gill slit appears tilted towards the right, and the opening emerges on the right side; the second gill slit forms in a more medial position. (MOV 730 KB)

Additional file 5: Video S2: A 3D model reconstructed from z-stack images of pharyngeal structures in a *B. floridae* larva treated with SB505124. The anterior is pointed towards the left side. The initial view of the larva is a lateral view, which tilts slightly towards the left side. The larva is rotated clockwise when viewed from the posterior end. The preoral pit does not form in the treated larva, leaving a vacant region within the rostrum. The mouth does not open in the treated larva. The endostyle (blue) forms ectopically on the left side, resulting in a ‘U’-shaped organ. The club-shaped gland (red), still posterior to the endostyle, also forms a ‘U’-shaped structure, caused by the ectopic formation of the organ on the left. The first two gill slits (purple) are both located medially in the treated larva. (MOV 644 KB)

## Abbreviations

BLAST: Basic Logical Alignment Search Tool
BMP: bone morphogenetic protein
CNS: central nervous system
DIG: digoxigenin
DMSO: dimethyl sulfoxide
EST: expressed sequence tag
LR: left-right
SELI: self-enhancement and lateral inhibition
tBLASTx: translated nucleotide BLAST
TGF-β: transforming growth factor beta.
