# Supplementary material for: The Nodal signaling pathway controls left-right asymmetric development in amphioxus
Source: EvoDevo. 2015 Feb 17;6:5. doi: 10.1186/2041-9139-6-5 (PMC4423147; doi:10.1186/2041-9139-6-5)
Supplement: Supplementary file 1 — Additional file 1: Table S1: List of clones from the EST library used to synthesize probes of B. floridae genes. (DOCX 42 KB) [file 13227_2014_150_MOESM1_ESM.docx]

**Additional file 1:**

**Table S1. List of clones from EST library used to synthesize probes of *B. floridae* genes**

| Gene names | EST Clones |
| --- | --- |
| B.f. Gdf1/3 | bflv005f04 |
| B.f. Pitx | bfne159j14 |
| B.f. m-actin | bfne159i15 |
| B.f. Nkx2.1 | bfne062i09 |
| B.f. Hu/Elav | bfne128i05 |
| B.f. Hand | bfga046k20 |
| B.f. ERR | bfne065a22 |
