# Supplementary material for: The Nodal signaling pathway controls left-right asymmetric development in amphioxus
Source: EvoDevo. 2015 Feb 17;6:5. doi: 10.1186/2041-9139-6-5 (PMC4423147; doi:10.1186/2041-9139-6-5)
Supplement: Supplementary file 2 — Additional file 2: Table S2: List of PCR primers used for amplifying cDNA fragments of B. floridae and B. lanceolatum genes. (DOCX 97 KB) [file 13227_2014_150_MOESM2_ESM.docx]

**Additional file 2:**

**Table S2. List of PCR primers used for amplifying cDNA fragments of *B. floridae* and *B. lanceolatum* genes**

| Gene names | Forward Primer | Reverse Primer |
| --- | --- | --- |
| B.f. Cerberus | 5’-CTTGCGGCCGCTCACTATAGGGCGAATTGGGTACC-3’ | 5’-CGGGATCCCGGACAGGCGCACTCCTCTACT-3’ |
| B.f. genes from EST clones*: Gdf1/3, Pitx, m-actin, Nkx2.1, Hu/Elav, Hand | 5’-ATTTAGGTGACACTATAGAAGACGGCCAGTCTTAAGCTC-3’ | 5’-TAATACGACTCACTATAGGGAGGGGATATCAGCTGGATG-3’ |
| B.l. Pitx | 5’-GCTTGGACCAACCTCACAGAG-3’ | 5’-TCGACGAACTCTGAACAGCT-3’ |
| B.l. Lhx3 | 5’-CCAGCTGGATTCAACAATAG-3’ | 5’-TACGGACTGACTGTCTACCG-3’ |
| B.l. Dkk1/2/4 | 5’-AACGATGTCGATCTCCATGC-3’ | 5’-GCATTGGAACAATGATAGCC-3’ |
| B.l. FoxE4 | 5’-CTCCACGTACATGGGGAACT-3’ | 5’-TGTCATATCGGCGAGACGTA-3’ |
| B.l. FoxQ1 | 5’-TGAACGCGTCCTAAAACTCT-3’ | 5’-AACATCGCGATCCAAAAAGT-3’ |
| B.l. Nkx2.1 | 5’-CTAGTCAAAGACGGCAAGCCG-3’ | 5’-TGCTACAATACTGGCACGTCC-3’ |
| B.l. Krox | gift of S. Bertrand |  |

Footnotes: * These *B. floridae* cDNA fragments are in the pDONR222 vector (Invitrogen) and can be amplified by PCR using the listed primer set.
