# Supplementary figures and images for: The Nodal signaling pathway controls left-right asymmetric development in amphioxus
Source: EvoDevo. 2015 Feb 17;6:5. doi: 10.1186/2041-9139-6-5 (PMC4423147; doi:10.1186/2041-9139-6-5)

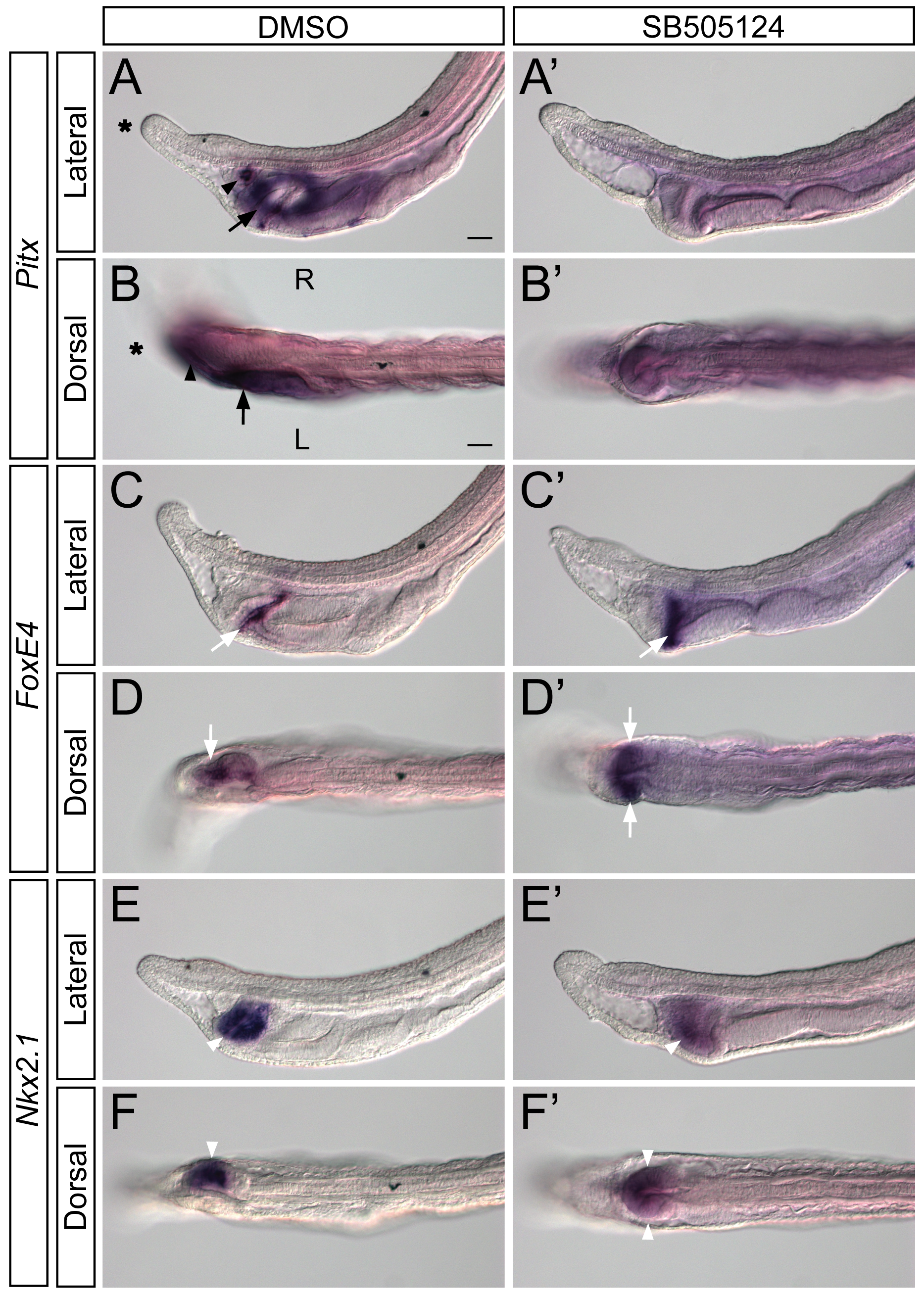

Supplement: Supplementary file 3 — Additional file 3: Figure S1: Branchiostoma floridae larvae display similar morphological changes upon treatment with SB505124. Asterisks (*) mark the anterior, ‘L’ marks the left side, and ‘R’ marks the right side. Scale bar, 25 μm. (A, A’, B, B’) Pitx expression marks the left-sided mouth (black arrow) and preoral pit (black arrowhead); both structures are lost upon treatment with SB505124. (C, C’, D, D’) FoxE4 expression marks the whole club-shaped gland (white arrow), which resides mainly on the right side of the larva. Upon treatment, the club-shaped gland appears symmetrically on both the left and right sides. (E, E’, F, F’) Nkx2.1 is expressed in the right-sided endostyle (white arrowhead). In the embryos treated with SB505124, the endostyle forms on both the left and right sides. (TIFF 9 MB) [file 13227_2014_150_MOESM3_ESM.tiff]
